# Time, Concentration, and pH-Dependent Transport and Uptake of Anthocyanins in a Human Gastric Epithelial (NCI-N87) Cell Line

**DOI:** 10.3390/ijms18020446

**Published:** 2017-02-18

**Authors:** Allison A. Atnip, Gregory T. Sigurdson, Joshua Bomser, M. Mónica Giusti

**Affiliations:** 1 Department of Food Science and Technology, The Ohio State University, Columbus, OH 43210, USA; Atnip.1@osu.edu (A.A.A.), Sigurdson.5@osu.edu (G.T.S.); 2 Department of Human Nutrition, The Ohio State University, Columbus, OH 43210, USA; Bomser.1@osu.edu

**Keywords:** anthocyanins, gastric, stomach, uptake, NCI-N87 cell line, chokeberry

## Abstract

Anthocyanins are the largest class of water soluble plant pigments and a common part of the human diet. They may have many potential health benefits, including antioxidant, anti-inflammatory, anti-cancer, and cardioprotective activities. However, anthocyanin metabolism is not well understood. Studies suggest that anthocyanins absorption may occur in the stomach, in which the acidic pH favors anthocyanin stability. A gastric epithelial cell line (NCI-N87) has been used to study the behavior of anthocyanins at a pH range of 3.0–7.4. This work examines the effects of time (0–3 h), concentration (50–1500 µM), and pH (3.0, 5.0, 7.4) on the transport and uptake of anthocyanins using NCI-N87 cells. Anthocyanins were transported from the apical to basolateral side of NCI-N87 cells in time and dose dependent manners. Over the treatment time of 3 h the rate of transport increased, especially with higher anthocyanin concentrations. The non-linear rate of transport may suggest an active mechanism for the transport of anthocyanins across the NCI-N87 monolayer. At apical pH 3.0, higher anthocyanin transport was observed compared to pH 5.0 and 7.4. Reduced transport of anthocyanins was found to occur at apical pH 5.0.

## 1. Introduction

Increased dietary intake of anthocyanins may be associated with improved health; therefore, there has recently been an increased interest in understanding anthocyanin metabolism in humans [1,2,3,4,5,6,7]. The complex chemistry, dynamic stability, and behavioral variations of anthocyanins all contribute to the difficulty of the study of anthocyanin uptake, transport, bioavailability, and metabolism. Given their different structural forms at various pH levels and their susceptibility to degradation by pH, microbes, and enzymes, studies of the rates of uptake, metabolism, and cellular distributions of anthocyanins often give differing results. In order to investigate the role of these compounds in disease prevention, determining the details of their transport and uptake is an initial key step. Therefore, the effects of time and initial concentration on the transport and uptake of chokeberry anthocyanins by NCI-N87 cells were investigated here.

Anthocyanins comprise a subgroup of flavonoid compounds which are characterized by a C_6_C_3_C_6_ skeletal backbone. The basic structure of the aglycone is that of an aromatic ring (A ring) bound to a heterocyclic, oxygen-containing ring (C ring), which is bound by C–C bond to another aromatic ring (B ring). Anthocyanins exist in a dynamic equilibrium in a solution that is profoundly dependent on environmental pH: under acidic conditions (pH 1.0–3.5) the flavylium cation is the most dominant form, and the color is relatively more stable. In mildly acidic conditions (pH 5–6), the open-ring chalcone or hemiketal forms predominate, and the compounds lose color. In alkaline conditions (pH ≥ 7), the quinoidal base is dominant, and unstable purple-blue colors are formed [8]. This dynamic structural equilibria is a property that differentiates anthocyanins from the rest of the flavonoid family and has consequences on the transport of anthocyanins throughout the gastrointestinal tract [9].

Many studies have shown that anthocyanins are poorly absorbed compared to other flavonols; absorption of less than >1% of ingested anthocyanins has been shown by various animal studies [10,11,12]. Additionally, the majority of ingested anthocyanin has been shown to disappear from the GI tract within 4 h [9]. Although the stomach is mostly regarded as a storage organ where some mixing and chemical digestion takes place, for anthocyanins, it seems to serve as a site of significant absorption. In the acidic pH common to the stomach, anthocyanins are stable flavylium cations; absorption here is most likely this form of the compounds [13]. Anthocyanins have been found to appear very quickly in the plasma of rats, along with high concentrations of anthocyanin metabolic products such as protocatechuic acid and 4-hydroxybenzoic acid [14]. When the stomachs of anesthetized rats were directly exposed to standardized mixtures of anthocyanins or berry extracts, about 25% of administered anthocyanin monoglycosides were rapidly absorbed, unmetabolized, regardless of the aglycone structure [2]. In human studies, intact anthocyanins have been detected in the plasma and urine following an oral dose after just 10 min [15].

Several other studies have also shown rapid appearance of anthocyanins in plasma upon ingestion (in both humans and rats), thereby suggesting significant gastric uptake [16,17,18,19,20]. It was hypothesized that anthocyanin uptake in the stomach is by bilitranslocase, a transport protein capable of interacting with many substrates [16,17,20]. Bilitranslocase is present in both the liver and gastric epithelium, in similar forms. Via a bilitranslocase transport activity assay in rat plasma membrane vesicles, 17 of 20 tested anthocyanins acted as competitive inhibitors of bilitranslocase transport activity (*K*_I_ = 1.4–22 µM) [16]. Given their relative size and their charged cationic form in lower pH, passive diffusion through the gastric cells is unlikely.

Cell culture can be a useful tool to study the behavior of compounds in various tissues. However, most gastric epithelium cell lines must be maintained at physiological pH ~7.4, or the cell monolayer integrity will be lost or cells may even die [21]. This is a limitation regarding in vitro evaluation of anthocyanins, due to their pH dependent chemical equilibria. Gastric mucosa production by cell line models increases cellular stability to acidic pH. Some animal derived models exist; however, the only human derived gastric mucosa producing models are the MKN-28 and NCI-N87 lines [22]. With the MKN-28 cell line, anthocyanin transport was evaluated in pH 5.0 and 7.4 and was found to decrease with increased pH [22]. The NCI-N87 cell line offers more flexibility in terms of its environmental pH. The NCI-N87 cell line was established from human gastric carcinoma and forms a well-differentiated monolayer, which expresses epithelial-type junctions (E-cadherin and zonula occludens-1 expression) and post-confluency secretes gastric mucin-6 glycoprotein. At this stage the cell line is acid-resistant, having shown stability from pH 3.0–7.4 [23]. These factors make this cell line a physiologically relevant model of the gastric epithelium, so it has been used to investigate drug metabolism in the stomach [23,24,25]. This cell line was validated as an effective model to study the uptake and transport of anthocyanins in pH 3.0–7.4 and therefore may prove useful for investigating the uptake and metabolism of dietary flavonoids in the stomach [26]. This is the first known report to document the uptake and transport of anthocyanins at pH 3.0, and compare to other pH levels under the same system.

Several factors play into the metabolism of anthocyanins, and much of these remain to be explored. The objective of this work was to determine the effects of time, initial concentration, and apical pH (simulating the interior of the stomach) on the transport and uptake of anthocyanins by NCI-N87 gastric cells. These studies provide insight on the transportation of anthocyanin in the stomach as affected by the amount of anthocyanin and the time of interaction between the pigments and cells. The variations in apical pH will simulate fasted and fed state pH conditions in the stomach and will result in the modification of the anthocyanin structural equilibria, thereby providing more information as to how these structures affect anthocyanin.

## 2. Results and Discussion

The NCI-N87 cell line was previously shown to be an effective model to evaluate the transport and uptake of various drugs by gastric tissues in pH 3.0–7.4 [26]. The cell line was evaluated for suitability to study the uptake and transport of anthocyanins in similar conditions prior to the analyses presented here. To summarize, the cell density of the monolayer was not affected by exposure to anthocyanin concentrations ≤1500 µM, evaluated by the Sulforhodamine B assay [26]. When seeded on inserts, the monolayer also maintained integrity in all treatment pH according the results of phenol red assays, with the exception of slight decrease that was rapidly recovered which occurred with treatment apical pH at 3.0. Further indications of active transport mechanisms were suggested by halted transport when cells were treated at 0 °C [26]. These findings demonstrated the NCI-N87 cell line to be a physiologically relevant model to study transport and uptake of anthocyanins by gastric tissue.

### 2.1. Effects of Time and Initial Concentration on the Transport and Uptake of Anthocyanins by NCI-N87 Cells

**Figure 1:**
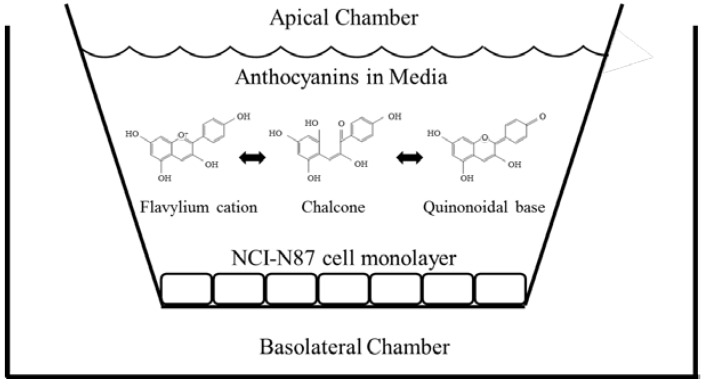
Representation of growing conditions of NCI-N87 cell line and treatments with anthocyanins at different pH conditions.

Chokeberry extract (pH 3.0) was added to the apical side of the cell monolayer at varying concentrations (50–1500 µM). Aliquots of the basolateral layer (pH 7.4) were removed at the desired timepoints (15–180 min) (see Figure 1). The apical, basolateral, and cell layers were collected after 180 min and analyzed by High Pressure Liquid Chromatography-Photo Diode Array-Mass Spectrometry (HPLC-PDA-MS).

**Figure 2:**
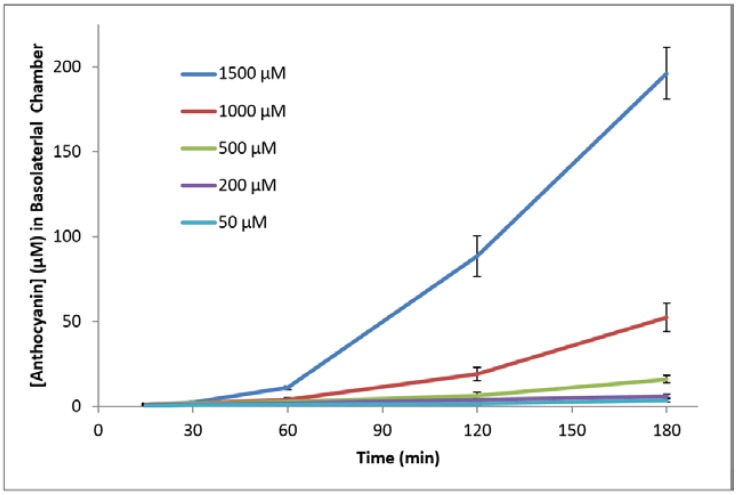
Effect of time on the concentration (µM) of anthocyanins transported through the NCI-N87 monolayer (apical to basolateral chambers) at apical pH 3.0, with varying initial concentrations over 3 h at 37 °C.

Anthocyanins were detected in the basolateral chamber in 15 min with initial concentrations >200 µM (1.0 and 1.4 µM anthocyanin concentrations detected for 500 and 1500 µM initial concentrations, respectively). However, anthocyanins were not detected in the basolateral chamber at 15 min with the initial concentration of 50 µM. At 120 min, significant differences in the anthocyanin concentrations in the basolateral chamber anthocyanin concentrations were found between 1500, 1000, and 500 µM treatment concentrations, respectively, with 88.7 > 19.2 > 6.4 µM detected (*p* < 0.05). At 180 min, significant differences were observed between all initial concentrations: 3.7, 6.2, 16.2, 52.6, and 196.2 µM in the basolateral chamber at 50, 200, 500, 1000, and 1500 µM initial concentration, respectively (*p* < 0.05). The concentration of anthocyanins transported from the apical to basolateral chamber increased over time (see Figure 2).

The rate of transport over time was non-linear (see Figure 2), which was especially observable with higher apical concentrations of 1000 and 1500 µM. This suggests increased activity by a possible active transport mechanism, in agreement with previous literature [16,18,19,27]. Transport by passive diffusion alone would have resulted in an approximately linear relationship between time and the basolateral concentration. The transport of anthocyanins did not plateau even with the highest anthocyanin concentration (1500 µM) at 180 min (see Figure 2). This suggests that conditions of this study did not saturate the mechanism of transport or the possible transport protein. Further evaluations would be necessary to determine conditions to plateau the transport of anthocyanins through the NCI-N87 monolayer.

The rapid appearance of anthocyanins in the basolateral chamber (15 min), similarly to previous reports [16,17,18,19,20], suggests that gastric uptake and transport may be significant steps in the metabolism of anthocyanins. The time-dependent transport of anthocyanins in the stomach should be considered in the use of dietary anthocyanins for health.

**Figure 3:**
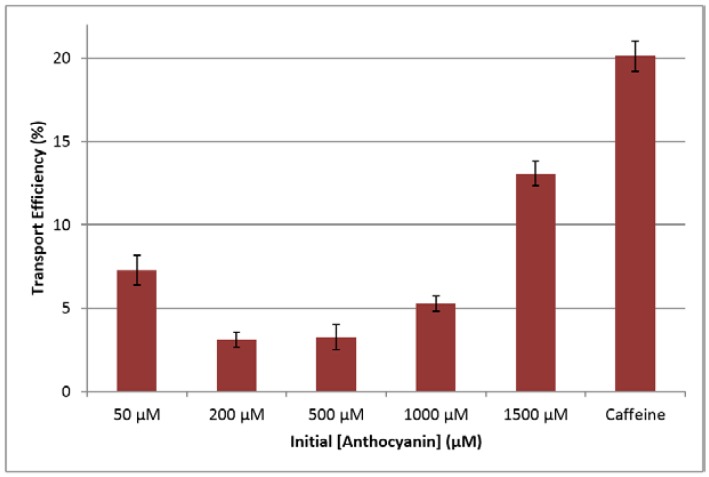
Effect of initial anthocyanin concentration on the transport efficiency (%) of anthocyanins through the NCI-N87 monolayer over 3 h at 37 °C at apical pH 3.0 and basolateral pH 7.4. Initial caffeine concentration was 1500 µM. Transport efficiency was determined by ([Anthocyanin] in basolateral chamber/initial apical [Anthocyanin]) × 100.

The initial apical concentration of 50 µM showed significantly higher transport efficiency than both 200 and 500 µM concentrations (*p* < 0.05), but the initial concentration of 1500 µM showed significantly higher transport efficiency than all other initial concentrations. Interestingly, the transport efficiency of the initial concentration of 1000 µM was not significantly different than 50 µM. Generally, concentrations in the basolateral side increased with time and concentration (see Figure 3).

The transport efficiencies of anthocyanin treatments were compared to that of caffeine, known to diffuse passively through the gastric monolayer [23]. Caffeine showed significantly higher transport efficiency than the anthocyanin treatments, thereby providing further evidence for active transport mechanisms for anthocyanins. The higher transport efficiency of low concentrations (50 µM) of anthocyanins compared to the larger concentrations suggests this cell line has a strong preference and potential use for these compounds. With higher concentrations, the transport efficiency decreased as similar or higher amounts of the compounds could be transported with less effort.

**Figure 4:**
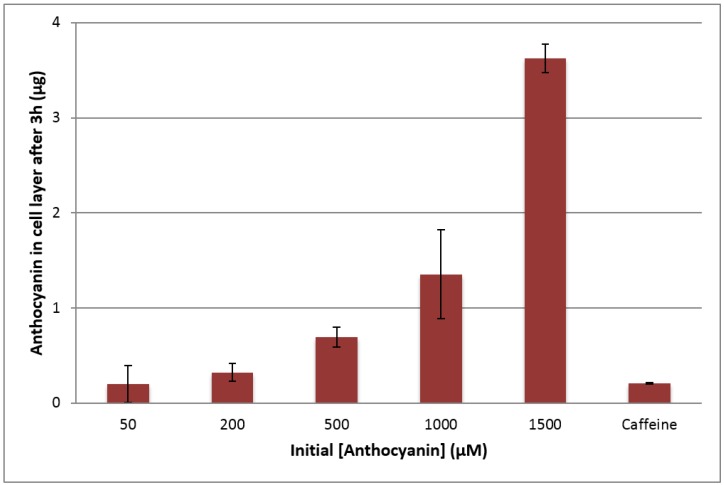
Effect of initial anthocyanin concentration (µM) on the total anthocyanin amount (µg) extracted from the NCI-N87 cell layer after 3 h treatment at 37 °C at apical pH 3.0 and basolateral pH 7.4. Initial caffeine concentration was 1500 µM.

The amounts of anthocyanins taken up by the cells were also analyzed by collecting the cell layers after treatment. More anthocyanins were extracted from the cells with each increase in the initial concentration of 50, 200, 500, 1000, and 1500 µM (see Figure 4), significantly increasing with each increase in initial anthocyanin concentration (*p* < 0.05). With low initial 50 µM anthocyanin concentration treatments, anthocyanins were not always detected in cells for every treatment replicate, accounting for the large standard deviation of this data set. Caffeine was again used as a control for passive diffusion through the gastric monolayer; it is not necessarily taken up or metabolized by gastric cells [28]. Despite the high initial concentration of caffeine (1500 µM), the amount of caffeine extracted from the cells was relatively low, and was statistically similar to the initial anthocyanin concentration of 50 µM.

At 50 µM initial anthocyanin concentration, uptake efficiency was higher than that observed at 200, 500, and 1000 µM. Highest uptake efficiency was observed at 1500 µM (*p* < 0.05). Despite the large standard deviation associated with the 50 µM uptake efficiency due to extremely low anthocyanin amounts and the limits of detection of the instruments, efficiency was still calculated to be significantly higher (0.41%) than the uptake efficiencies for 200, 500, and 1000 µM (0.17%, 0.14%, and 0.14%, respectively). Interestingly, the uptake efficiency at 1500 µM (0.25%) was not significantly different than that of 50 µM. Anthocyanin uptake efficiency exhibited similar trends as transport efficiency, and neither followed linear dose-dependent responses.

There is convincing evidence supporting anthocyanin transport through the gastric epithelium; however, data on the cellular uptake or accumulation of anthocyanins is limited. In rats fed a diet high in blueberry anthocyanins (14.8 mmol/kg for 15 days), 62.9 nmol anthocyanins/g tissue were found to accumulate in stomach tissue [2]. After rats were administered with black raspberry anthocyanins, red color was also observed in gastric tissue samples [29]. Presence of anthocyanins was confirmed by observing spectroscopic changes at pH 1.0, 4.5, and 10, and the pigments appeared to be irreversibly bound to tissue [29]. Therefore, the anthocyanins could not be quantified as free anthocyanins, and the researchers hypothesized that the anthocyanins may be bound to the transporter protein responsible for gastric uptake [29]. In another study, color in some cell samples was observed after multiple extractions (with efficiency of approximately 85%), thereby further indicating possible protein binding [30].

### 2.2. Effects of pH on the Recovery and Transport and Uptake of Anthocyanins by NCI-N87 Cells

To model some of the in vivo gastric pH conditions that anthocyanins may encounter after ingestion, the apical pH was adjusted to pH 3.0, pH 5.0, or pH 7.4 to evaluate the effects of anthocyanin structures on uptake and transport. Basolateral pH was maintained at physiological pH 7.4. The pH in the stomach can be less than 3; however, the NCI-N87 cell monolayer has been shown to lose its transepithelial electrical resistance and thus, monolayer integrity. The stability of anthocyanins is known to be affected strongly by environmental pH, typically decreasing as pH is increased. Therefore, it can be proposed that the gastric pH may play a role in the stability of anthocyanins as well as their transport and uptake. [23].

Apical pH of 3.0 provided the highest total anthocyanin recovery (91.7% ± 3.2%) (see Table 1). Total anthocyanin recovery at pH 5.0 was 81.7% ± 4.4%, less than at pH 3.0. Recovery at pH 7.4 was measured as 75.4% ± 4.1%, less than both at pH 3.0 and pH at 5.0. There was over a 10% difference in recovery from the apical chamber between treatment at pH 3.0 and 7.4, thereby showing that pH did have an important effect on the anthocyanin structural equilibrium in solution. The more stable flavylium cation form of the anthocyanin predominates at pH 3.0; while at pH 5.0, the hydrated chalcone form of the anthocyanin dominates, which is more prone to breakdown.

Interestingly, these same trends were not observed from the anthocyanins received from the cell layers. At pH 3.0, recovery from the cells was 0.9% ± 0.1%, significantly lower than at pH 5.0, which showed the highest cellular recovery at 1.3% ± 0.1%. Recovery from the cell layer at pH 7.4 was the lowest at 0.6% ± 0.0%. Cellular uptake of anthocyanins also seemed to be affected by the anthocyanin structures which are affected by pH.

**Figure 5:**
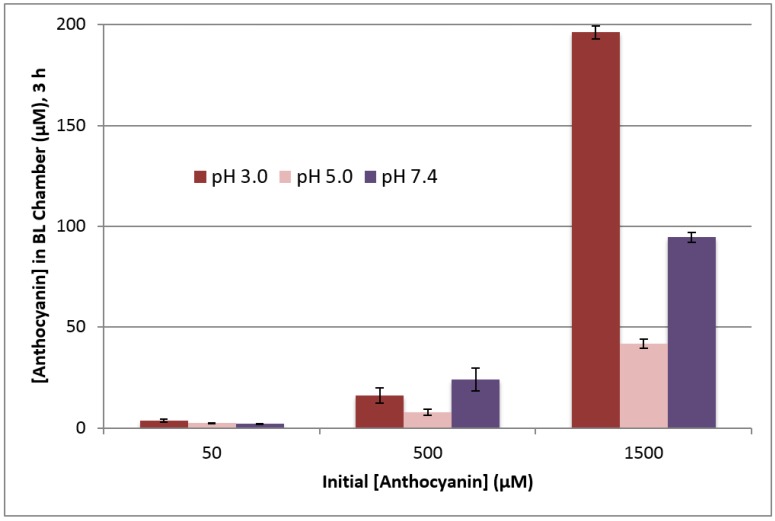
Effect of apical pH on the transport of chokeberry anthocyanins through the NCI-N87 cell monolayer after 3 h at 37 °C and basolateral pH 7.4 as determined by anthocyanin concentration in basolateral chamber.

With change in anthocyanin concentration in the apical chamber, each pH level showed an overall dose dependent response; however, the effects were not uniform over all pH and concentration levels (Figure 5). At pH 3.0, the anthocyanin concentration in the basolateral chamber was significantly higher with initial apical concentrations of 50 and 1500 µM compared to the other pH levels at the same concentrations (see Table 2). At 50 µM, pH 5.0 and pH 7.4 were not significantly different. At 1500 µM, pH 3.0 was significantly higher than both other pH levels, followed by pH 7.4, and pH 5.0 was the lowest transport at the higher concentration (see Table 2) (*p* < 0.05). However, with initial concentrations of 500 µM, the anthocyanin transports for the pH 3.0 and 7.4 treatments were not significantly different from each other; however, both were significantly higher than pH 5.0 in which anthocyanins exist predominantly in open-ring formations. As the transport of anthocyanins across the monolayer is most likely via active transport, the interaction between anthocyanin and the transport protein would likely be affected by this change in structure.

At apical pH 3.0 and 7.4, the concentration of transported anthocyanins was greater than at pH 5.0, where hydrated and open ring anthocyanins predominate; this suggests closed ring anthocyanins may be preferentially transported. At pH 3.0 the flavylium cation is the predominant anthocyanin structure; these results suggest that this form may be preferentially transported through the NCI-N87 cell monolayer. At pH 7.4, quinoidal bases are formed and like the flavylium cations, these are also closed ring structures. These findings suggest that pH may not only play a significant role in the stability of anthocyanins in various possible gastric environments, but also in the transport of anthocyanins (see Figure 5).

Treatments with 50 and 1500 µM anthocyanins at pH 3.0 resulted in the highest transport efficiency of all experiments (7.31% ± 0.21% and 13.08% ± 0.73%, respectively). Apical pH 5.0 resulted in significantly lower transport efficiency with all treatment concentrations (4.75% ± 0.48%, 1.57% ± 0.06%, and 2.79% ± 0.10% for initial concentrations of 50, 500, and 1500 µM respectively; *p* < 0.05). Interestingly, at the initial concentration of 500 µM, the pH 3.0 and 7.4 treatments were not statistically different from each other (3.24% ± 0.02% and 4.83% ± 1.14% respectively), nor were the 50 and 500 µM treatments at pH 7.4 (4.05% ± 0.31% and 4.83% ±1.14%, respectively). Transport efficiency followed similar patterns observed for concentrations of transported anthocyanins.

**Figure 6:**
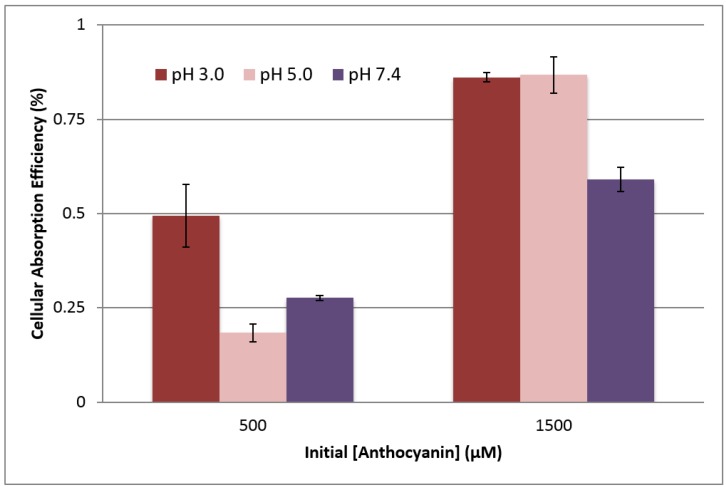
Effects of apical pH on the cellular anthocyanin uptake efficiency (%) of NCI-N87 cells after 3 h treatment at 37 °C and basolateral pH 7.4. Uptake efficiency was calculated as (Concentration extracted from cell layer/Initial treatment concentration) × 100.

Apical pH again played a role in anthocyanin uptake (see Figure 6). At the apical concentration of 50 µM, the anthocyanin content in the cell layer was almost impossible to detect under the analytical conditions used. With 500 µM apical treatments, pH 3.0 showed significantly higher uptake efficiency (0.49% ± 0.08%) as compared to both pH 5.0 and 7.4 (0.28% ± 0.01% and 0.18% ± 0.02%, respectively). This suggests that the cell may preferentially take up the flavylium cation form of the anthocyanin. However, at the higher initial anthocyanin concentration of 1500 µM, the uptake at pH 3.0 was not significantly different than that at pH 5 (0.86% ± 0.01% and 0.87% ± 0.05%, respectively). Both of these pH levels were significantly higher than uptake at pH 7.4 (0.59% ± 0.03%) at this concentration. With increases in concentration, anthocyanins can undergo self-association; this stacking of the molecules leads to increased stability and color expression [31]. The association of these different molecules reduces hydration of the chromophore and therefore decreases the formation of the open-ring chalcone. This chemistry may have played a role in the lack of a statistically significant difference between cellular uptake with 1500 µM treatments at pH 3.0 and 5.0. The lower uptake at pH 7.4 may indicate that the NCI-N87 cells less preferentially take up the quinoidal base form of the anthocyanin. It may also be the result of the greater overall anthocyanin loss at pH 7.4 (Table 1), as anthocyanins are generally less stable at this pH. The increased cellular uptake at pH 5.0 with the high anthocyanin concentration (1500 µM) treatment may also suggest a change in the uptake mechanism by the cells themselves, possibly triggered by high concentrations.

## 3. Materials and Methods

Unless specifically indicated, all supplies and chemicals were purchased from Sigma Aldrich (St. Louis, MO, USA) and Fisher Scientific (Pittsburgh, PA, USA). Anthocyanin extracts were prepared from chokeberry juice concentrate donated by Artemis International (Fort Wayne, IN, USA). Extracts were stored under dark freezing (−20 °C) conditions.

### 3.1. Extraction and Purification of Anthocyanins for Cell Culture Assays

Chokeberry juice concentrate was obtained (courtesy of Artemis International (Fort Wayne, IN, USA)) and stored under dark refrigeration (4 °C) conditions. The anthocyanin-rich chokeberry extract was prepared by the methods of Rodriguez-Saona and Wrolstad and He and Giusti [32,33]. Briefly, the juice concentrate was extracted with an equal volume of acetone and filtered through Whatman #1 paper. Hydrophobic compounds were partitioned from the acetone extract by addition of two parts chloroform to one part acetone filtrate (*v*/*v*) and storage overnight at 4 °C. The aqueous phase was collected and residual acetone or chloroform was removed by rotary evaporation. The resulting material was then purified by cation exchange (MCX SPE cartridge, Waters Corp., Milford, MA, USA). Neutral and anionic phenolics were removed with 0.1% trifluoroacetic acid in water and then 0.1% trifluoroacetic acid in methanol. Anthocyanins were then eluted into a flask containing 500 µL 88% formic acid with water:methanol 40:60 *v*/*v* with 1% NH_4_OH followed by methanol with 1% NH_4_OH. The anthocyanin content of the extract was determined by High Pressure Liquid Chromatography (HPLC), as described below.

### 3.2. Cell Culture Conditions

The gastric epithelial cell line NCI-N87 is derived from human gastric carcinoma tissue and was purchased from the American Type Culture Collection (Manassas, VA, USA). Cells were initially seeded in 75-cm^2^ culture flasks (Corning, Corning, NY, USA) and maintained with Roswell Park Memorial Institute-1640 complete medium supplemented with 10% fetal bovine serum, 100 µg/mL penicillin, and 100 µg/mL streptomycin. Cells were grown at 37 °C under 5% CO_2_. Seeding on plastic and on inserts was at a density of 2.5 × 10^5^. All experiments were performed on passages 4–30.

### 3.3. Effects of Concentration and Time on the Transport of Anthocyanins through the NCI-N87 Monolayer

For these treatments, chokeberry extract was mixed with Hank’s Buffered Salt Solution (HBSS) to obtain the desired anthocyanin concentrations (50–1500 µM) and adjusted to pH 3.0 with 6.0 N HCl. Growth media was removed from the apical and basolateral chambers, and they were washed with HBSS. The different concentrations of diluted chokeberry anthocyanins at pH 3.0 were added to the apical chambers, and the basolateral chambers were refilled with new pH 7.4 HBSS. Aliquots from the basolateral chambers were removed at time points of 15, 30, 60, 120, and 180 min, and the difference was replaced with pH 7.4 HBSS. After 180 min, apical and basolateral samples and cell layers were collected. Samples were prepared and analyzed by HPLC-MS. These experiments were conducted in triplicate.

### 3.4. Effects of pH on the Transport of Anthocyanins through the NCI-N87 Monolayer

To simulate fed and fasted states in the stomach, HBSS was adjusted to the desired pH (3.0, 5.0, 7.4) with 6.0 N HCl and 1.0 N NaOH, not exceeding 0.5% of the total treatment solution, after mixing with purified chokeberry anthocyanins to obtain treatment concentrations (50–1500 µM). HBSS pH 7.4 was placed in the basolateral chambers, and the anthocyanin pH concentration treatment solutions were added to apical chambers. Apical pH was monitored throughout the 180 min treatment to ensure stability and consistency. Treatments at apical pH 3.0 were prone to slightly increase during treatment time [26]. To maintain consistent pH 3.0, small aliquots of 6 N HCl were added at 30 and 120 min time points, similarly employed on the MKN-28 gastric cell line [27]. The total sum of HCl added to the media from initial pH adjustment and during treatment was ≤0.3% HCl. After 180 min with treatment at 37 °C and 5% CO_2_, the apical, basolateral, and cell layers were collected and analyzed by HPLC-MS.

### 3.5. Apical and Basolateral Sample Preparation for HPLC Analysis

After treatment, spent media was collected and immediately mixed 1:1 (*v*/*v*) with acidified (1% HCl) methanol. Mucus layers were removed by quick-washing 3× with cold phosphate-buffered saline (PBS). Apical and basolateral samples were vortexed, centrifuged. The supernatant from each was filtered through 0.2 µm nylon syringe filters for HPLC analysis. Samples were stored in a dark −20 °C freezer until analysis; however, when possible analysis was conducted immediately after collection.

### 3.6. Anthocyanin Extraction from NCI-N87 Cells

Cell samples were collected via cell lifter in phosphate-buffered saline (PBS) and centrifuged. This PBS was removed, and 0.75 mL of new PBS was added to the cells, which were then lysed and homogenized by sonication. Next, 0.75 mL of extraction solution (70:25:5 acetone:water:formic acid *v*/*v*) was added and the samples were frozen overnight. After thawing, samples were again sonicated, vortexed, and centrifuged (14,000 rpm, 5 min, 0 °C). Supernatants were then collected into carefully pre-weighed glass tubes. Extraction was repeated twice more, without overnight freezing. Supernatants were partially dried under N_2_ (30–45 min, 37 °C), weighed, and filtered (0.2 µm) for HPLC analysis. Density of samples was approximated to be 1 mg/mL by careful weighing and pipet measurement.

### 3.7. HPLC-PDA-MS Analysis of Samples

All samples were analyzed by HPLC under the same conditions. Liquid chromatography and mass spectrometry were performed using a Shimadzu HPLC-PDA-MS system (Columbia, MD, USA) equipped with LC-20AD pumps, SIL-20AC auto sampler, CTA-20A column oven coupled to an LCMS-2010 mass spectrometer, and SPD-M20A photodiode array detector. Data was collected and analyzed with LCMS Solution Software (Version 3.0, Shimadzu). Anthocyanin separation was achieved on a Symmetry C18 reverse-phase column (4.6 mm × 75 mm, 3.5 µm; Waters Corp., Milford, MA, USA) fitted with an analytical guard column (NovaPak^®^, 4 mm × 2.0 mm C18). The mobile phases were (A) 4.5% formic acid and (B) acetonitrile. A binary solvent gradient for chokeberry anthocyanin analysis followed: 0–3 min, 5%–10% B; 3–10 min, 10%–20% B, 10–15 min, 20%–35% B; 15–20 min, 35% B. Spectral data was collected from 250–700 nm. Mass spectrometry was performed under positive ion mode. Data were monitored using total ion scan (SCAN) (from *m*/*z* 100–1200), and selected ion monitoring was conducted for *m*/*z* of 271, 287, 301, 303, 317, and 331 representing common anthocyanidins.

Samples were quantified by external standard via construction of a five-point standard curve with cyanidin-3-glucoside (0–1500 µM) and area under the curve (AUC) with detection at 520 nm.

### 3.8. Statistical Analysis

Statistical analysis was performed in Microsoft Excel 2010 (Office 14.0, Microsoft, Redmond, WA, USA). Paired *t*-tests were used to obtain *p*-values to determine statistical significance. *p*-values of 0.05 or less were considered significant. Unless otherwise noted, all experiments were *n* = 6 and each sample was performed in duplicate.

## 4. Conclusions

This work showed evidence of the effects of time, concentration, and pH in an in vitro model of the gastric epithelium. Anthocyanins were transported through the NCI-N87 cell monolayer and taken up by the cells, in a time and concentration-dependent manner. Anthocyanins were increasingly absorbed as time of contact continued and as concentration was increased. Exposure of this gastric model to low concentrations of anthocyanins also showed high transport efficiency of these compounds which decreased as the anthocyanin concentration increased to a point. Findings from this work add support to the previous hypothesis that anthocyanins are transported through gastric tissue by means of active transport mechanisms.

The human stomach is subject to a wide range of pH levels, depending on an individual’s fed or fasted state, and can vary between individuals as well. Thus, any discussion of uptake or transport of compounds by the stomach must take into account its ever-changing environment. The NCI-N87 cell line provided a novel tool as an in vitro model to simulate the changing pH environment of the stomach. Environmental pH is known to have profound effects on the chemistry of anthocyanins in solution, and this work showed that pH also had a significant impact on the transport and uptake of anthocyanins by NCI-N87 cells. The flavylium cation, predominant in acidic pH, appeared to have been more preferentially taken up and transported by this cell line. While the chalcone form of the anthocyanin, with its open-ring structure, was generally less favorably transported by the cells. However, high concentrations of anthocyanins yielded similar cellular uptakes between pH 3.0 and 5.0, which was perhaps related to anthocyanin chemistry or an additional method of transport.

Some aspects of anthocyanin structure likely played a role in transport and uptake; however, given the vast amount of structural differences amongst anthocyanins in nature, much more work in this area is needed. This work did shed light on the basic question of possible impacts of structure on the transport and uptake of anthocyanins in the stomach. More work is needed to elucidate the effects of B-ring substitution, and various amounts and structures of glycosyl attachments as well as acyl moieties on anthocyanin transport and uptake in this model.

## Figures and Tables

**Table 1:** Average % anthocyanin recovery (SD: standard deviation) from apical, basolateral, and cell layers at varying apical pH values after 3 h treatment at 37 °C with initial anthocyanin concentration of 1500 µM.

Recovery Site	pH 3.0 (SD)	pH 5.0 (SD)	pH 7.4 (SD)
Apical	78.6 (2.6)	77.6 (4.3)	68.5 (2.9)
Basolateral	12.2 (0.9)	2.8 (0.4)	6.3 (0.1)
Cell	0.9 (0.0)	1.3 (0.1)	0.6 (0.0)
Total	91.7 (3.2)	81.7 (4.4)	75.4 (4.1)

**Table 2:** Anthocyanin concentrations (µM) collected from basolateral chambers with varying apical pH values after 3 h treatment at 37 °C.

Apical Concentration	pH 3.0 (SD)	pH 5.0 (SD)	pH 7.4 (SD)
50 µM	3.65 (0.77)	2.38 (0.10)	2.03 (0.15)
500 µM	16.22 (3.78)	7.83 (1.48)	24.17 (5.72)
1500 µM	196.25 (3.25)	41.83 (2.15)	94.55 (2.33)

## References

[1] He J., Magnuson B.A., Giusti M.M. (2005). Analysis of anthocyanins in rat intestinal contents—Impact of anthocyanin chemical structure on fecal excretion. J. Agric. Food Chem..

[2] Talavera S., Felgines C., Texier O., Besson C., Gil-Izquierdo A., Lamaison J.-L., Remesy C. (2005). Anthocyanin Metabolism in Rats and Their Distribution to Digestive Area, Kidney, and Brain. J. Agric. Food Chem..

[3] Kay C.D., Mazza G.J., Holub B.J. (2005). Anthocyanins exist in the circulation primarily as metabolites in adult men. J. Nutr..

[4] Kay C.D. (2006). Aspects of anthocyanin absorption, metabolism and pharmacokinetics in humans. Nutr. Res. Rev..

[5] Jing P., Bomser J.A., Schwartz S.J., He J., Magnuson B.A., Giusti M.M. (2008). Structure-Function Relantionships of Anthocyanidins from Various Anthocyanidin-Rich Extracts on the Inhibition of Colon Cancer Cell Growth. J. Agric. Food Chem..

[6] Nurmi T., Mursu J., Heinonen M., Nurmi A., Hiltunen R., Voutilainen S. (2009). Metabolism of berry anthocyanins to phenolic acidsin humans. J. Agric. Food Chem..

[7] Novotny J.A., Clevidence B.A., Kurilich A.C. (2012). Anthocyanin kinetics are dependent on anthocyanin structure. Br. J. Nutr..

[8] Castañeda-Ovando A., Pacheco-Hernández M.D.L., Páez-Hernández M.E., Rodríguez J.A., Galán-Vidal C.A. (2009). Chemical studies of anthocyanins: A review. Food Chem..

[9] Prior R.L., Wu X. (2006). Anthocyanins: Structural characteristics that result in unique metabolic patterns and biological activities. Free Radic. Res..

[10] Manach C., Williamson G., Morand C., Scalbert A., Remesy C. (2005). Bioavailability and bioefficacy of polyphenols in humans. I. Review of 97 bioavailability studies. Am. J. Clin. Nutr..

[11] Stoner G.D., Sardo C., Apseloff G., Mullet D., Wargo W., Pound V., Singh A., Sanders J., Aziz R., Casto B. (2005). Pharmacokinetics of anthocyanins and ellagic acid in healthy volunteers fed freeze-dried black raspberries daily for 7 days. J. Clin. Pharmacol..

[12] McGhie T.K., Walton M.C., Barnett L.E., Vather R., Martin H., Au J., Alspach P.A., Booth C.L., Kruger M.C. (2007). Boysenberry and blackcurrant drinks increased the plasma antioxidant capacity in an elderly population but had little effect on other markers of oxidative stress. J. Sci. Food Agric..

[13] Fang J. (2014). Some anthocyanins could be efficiently absorbed across the gastrointestinal mucosa: Extensive presystemic metabolism reduces apparent bioavailability. J. Agric. Food Chem..

[14] Tsuda T., Horio F., Osawa T. (1999). Absorption and metabolism of cyanidin 3-*O*-β-d-glucoside in rats. FEBS Lett..

[15] Milbury P.E., Cao G., Prior R.L., Blumberg J. (2002). Bioavailablility of elderberry anthocyanins. Mech. Ageing Dev..

[16] Passamonti S., Vrhovsek U., Mattivi F. (2002). The interaction of anthocyanins with bilitranslocase. Biochem. Biophys. Res. Commun..

[17] Passamonti S., Battiston L., Sottocasa G.L. (2000). Gastric uptake of nicotinic acid by bilitranslocase. FEBS Lett..

[18] Passamonti S., Vrhovsek U., Vanzo A., Mattivi F. (2003). The stomach as a site for anthocyanins absorption from food. FEBS Lett..

[19] Passamonti S., Terdoslavich M., Margon A., Cocolo A., Medic N., Micali F., Decorti G., Franko M. (2005). Uptake of bilirubin into HepG2 cells assayed by thermal lens spectroscopy: Function of bilitranslocase. FEBS J..

[20] Passamonti S., Vanzo A., Vrhovsek U., Terdoslavich M., Cocolo A., Decorti G., Mattivi F. (2005). Hepatic uptake of grape anthocyanins and the role of bilitranslocase. Food Res. Int..

[21] Phelan K., May K.M. (2015). Basic Techniques in Mammalian Cell Tissue Culture. Curr. Protoc. Cell Biol..

[22] Oliveira H., Fernandes I., Brás N.F., Faria A., de Freitas V., Calhau C., Mateus N. (2015). Experimental and Theoretical Data on the Mechanism by Which Red Wine Anthocyanins Are Transported through a Human MKN-28 Gastric Cell Model. J. Agric. Food Chem..

[23] Lemieux M., Bouchard F., Gosselin P., Paquin J., Mateescu M.A. (2011). The NCI-N87 cell line as a gastric epithelial barrier model for drug permeability assay. Biochem. Biophys. Res. Commun..

[24] Park J.G., Frucht H., LaRocca R.V., Bliss D.P., Kurita Y., Chen T.R., Henslee J.G., Trepel J.B., Jensen R.T., Johnson B.E. (1990). Characteristics of cell lines established from human gastric carcinoma. Cancer Res..

[25] Chailler P., Ménard D. (2005). Establishment of Human Gastric Epithelial (HGE) cell lines exhibiting barrier function, progenitor, and prezymogenic characteristics. J. Cell. Physiol..

[26] Atnip A., Sigurdson G.T., Giusti M.M., Bomser J.A. (2017). The NCI-N87 Cell Line as a Gastric Epithelial Model to Study Transport and Uptake of Anthocyanins. Food Funct..

[27] Fernandes I., de Freitas V., Reis C., Mateus N. (2012). A new approach on the gastric absorption of anthocyanins. Food Funct..

[28] Liguori A., Hughes J.R., Grass J.A. (1997). Absorption and subjective effects of caffeine from coffee, cola and capsules. Pharmacol. Biochem. Behav..

[29] He J., Wallace T.C., Keatley K.E., Failla M.L., Giusti M.M. (2009). Stability of Black Raspberry Anthocyanins in the Digestive Tract Lumen and Transport Efficiency into Gastric and Small Intestinal Tissues in the Rat. J. Agric. Food Chem..

[30] Kamonpatana K., Giusti M.M., Chitchumroonchokchai C., Morenocruz M., Riedl K.M., Kumar P., Failla M.L. (2012). Susceptibility of anthocyanins to ex vivo degradation in human saliva. Food Chem..

[31] Cavalcanti R.N., Santos D.T., Meireles M.A. (2011). A Non-thermal stabilization mechanisms of anthocyanins in model and food systems—An overview. Food Res. Int..

[32] Rodríguez-Saona L.E., Wrolstad R.E., Wrolstad R.E., Acree T.E., An H., Decker E.A., Penner M.H., Reid D.S., Schwartz S.J., Shoemaker C.F., Sporns P. (2001). Extraction, Isolation, and Purifification of Anthocyanins. Current Protocols in Food Analytical Chemistry.

[33] He J., Giusti M.M. (2011). High-purity isolation of anthocyanins mixtures from fruits and vegetables—A novel solid-phase extraction method using mixed mode cation-exchange chromatography. J. Chromatogr. A.

